# Effect of dietary nitrate on human muscle power: a systematic review and individual participant data meta-analysis

**DOI:** 10.1186/s12970-021-00463-z

**Published:** 2021-10-09

**Authors:** Andrew R. Coggan, Marissa N. Baranauskas, Rachel J. Hinrichs, Ziyue Liu, Stephen J. Carter

**Affiliations:** 1 Department of Kinesiology, Indiana University Purdue University Indianapolis, Indianapolis, IN 46202 USA; 2 Department of Kinesiology, Indiana University Purdue University Indianapolis, IF 101C, 250 University Boulevard, Indianapolis, IN 46112 USA; 3 Department of Kinesiology, Indiana University, Bloomington, IN 47405 USA; 4 University Library, Indiana University Purdue University Indianapolis, Indianapolis, IN 46202 USA; 5 Department of Biostatistics, Indiana University Purdue University Indianapolis, Indianapolis, IN 46202 USA; 6 Melvin and Bren Simon Comprehensive Cancer Center, Indiana University Purdue University Indianapolis, Indianapolis, IN 46202 USA

**Keywords:** Dietary nitrate, Nitric oxide, Muscle power, Humans, Individual participant data

## Abstract

**Background:**

Previous narrative reviews have concluded that dietary nitrate (NO_3_^−^) improves maximal neuromuscular power in humans. This conclusion, however, was based on a limited number of studies, and no attempt has been made to quantify the exact magnitude of this beneficial effect. Such information would help ensure adequate statistical power in future studies and could help place the effects of dietary NO_3_^−^ on various aspects of exercise performance (i.e., endurance vs. strength vs. power) in better context. We therefore undertook a systematic review and individual participant data meta-analysis to quantify the effects of NO_3_^−^ supplementation on human muscle power.

**Methods:**

The literature was searched using a strategy developed by a health sciences librarian. Data sources included Medline Ovid, Embase, SPORTDiscus, Scopus, Clinicaltrials.gov, and Google Scholar. Studies were included if they used a randomized, double-blind, placebo-controlled, crossover experimental design to measure the effects of dietary NO_3_^−^ on maximal power during exercise in the non-fatigued state and the within-subject correlation could be determined from data in the published manuscript or obtained from the authors.

**Results:**

Nineteen studies of a total of 268 participants (218 men, 50 women) met the criteria for inclusion. The overall effect size (ES; Hedge’s g) calculated using a fixed effects model was 0.42 (95% confidence interval (CI) 0.29, 0.56; *p* = 6.310 × 10^− 11^). There was limited heterogeneity between studies (i.e., I^2^ = 22.79%, H^2^ = 1.30, *p* = 0.3460). The ES estimated using a random effects model was therefore similar (i.e., 0.45, 95% CI 0.30, 0.61; *p* = 1.064 × 10^− 9^). Sub-group analyses revealed no significant differences due to subject age, sex, or test modality (i.e., small vs. large muscle mass exercise). However, the ES in studies using an acute dose (i.e., 0.54, 95% CI 0.37, 0.71; *p* = 6.774 × 10^− 12^) was greater (*p* = 0.0211) than in studies using a multiple dose regimen (i.e., 0.22, 95% CI 0.01, 0.43; *p* = 0.003630).

**Conclusions:**

Acute or chronic dietary NO_3_^−^ intake significantly increases maximal muscle power in humans. The magnitude of this effect–on average, ~ 5%–is likely to be of considerable practical and clinical importance.

**Supplementary Information:**

The online version contains supplementary material available at 10.1186/s12970-021-00463-z.

## Background

In 2007, Larsen et al. [1] reported that the ingestion of dietary nitrate (NO_3_^−^) reduced the oxygen (O_2_) cost of submaximal cycle ergometer exercise. Since then, over 100 studies have investigated the effects of this intervention (usually in the form of concentrated beetroot juice (BRJ)) on physiological responses and performance during endurance activities. Based on such research, recent systematic reviews and meta-analyses have concluded that dietary NO_3_^−^ supplementation exerts a small-to-moderate ergogenic effect on endurance performance, at least in non-athletes and during open-ended, time-to-fatigue exercise tests [2–7]. The mechanism responsible for this improvement is still unclear, but it is presumably the result of the reduction of NO_3_^−^ to nitrite (NO_2_^−^) and then to nitric oxide (NO) via bacterial or mammalian nitroreductases [8]. The resultant increase in NO bioavailability then decreases O_2_ demand and enhances performance during sustained exercise, seemingly by improving mitochondrial coupling [9] and/or by reducing ATP turnover itself [10]. Other studies, however, have failed to find any effects of NO_3_^−^ supplementation on mitochondrial function in either mice [11] or humans [11, 12], leaving a decrease in ATP utilization, possibly at the cross-bridge level, as a more plausible mechanism. It should also be noted that the beneficial effects of NO_3_^−^ ingestion on performance seem to be much smaller to insignificant in endurance athletes and/or during time trial type efforts [2–7].

More recently, attention has shifted to the effects of dietary NO_3_^−^ supplementation on performance during strength- or sprint-type activities, i.e., during brief skeletal muscle activity wherein aerobic ATP production is minimal. As recently reviewed by Alvares et al. [13], dietary NO_3_^−^ supplementation seems to have only a trivial impact on maximal force output during isometric or low velocity isotonic or isokinetic muscle contractions, although it may enhance the number of such repetitions that can be completed before failure [13, 14]. Animal studies, however, have demonstrated that the primary effects of NO on the contractile properties of muscle are not on force but on *speed*, i.e., on the maximal velocity of shortening, and hence on maximal muscle *power*, i.e., the product of force and speed [15, 16]. Consistent with this concept, a prior narrative review in 2018 concluded that acute dietary NO_3_^−^ increases maximal muscular power in humans [17]. The potential for dietary NO_3_^−^ to improve neuromuscular power is highly relevant to both athletic and patient populations alike, as power is typically more important than strength in determining either performance in sports [18, 19] or the ability to perform normal activities of daily living (e.g., standing up from a chair, climbing stairs) [20, 21]. The conclusions of this review, however, were based on roughly a dozen studies, and a number of pertinent investigations have since been published. Furthermore, no previous attempt has been made to employ meta-analysis to *quantitatively* assess the effects of dietary NO_3_^−^ supplementation specifically on maximal muscle power in humans. Such information would be useful for ensuring adequate statistical power in future studies of this treatment and may help place the effects of dietary NO_3_^−^ on various aspects of exercise performance (i.e., endurance vs. strength vs. power) in better context relative to each other.

To date, nearly all studies evaluating the effects of dietary NO_3_^−^ on either aerobic or non-aerobic exercise performance have relied upon a crossover experimental design, in which each individual serves as their own control. This is widely considered to be the “gold standard” for evaluating the effects of interventions with a short time-course of action (e.g., dietary NO_3_^−^), as it minimizes random variability in treatment effects and thereby increases statistical power [22]. Yet, prior meta-analyses of the effects of dietary NO_3_^−^ on exercise performance have either treated such within-subject data as a between-subject comparison [2–7, 23] or have assumed a relatively low, fixed within-subject correlation (i.e., *r* = 0.5) between the placebo and NO_3_^−^ trials [13]. Although common, these approaches are widely known to potentially distort the results of meta-analyses and possibly lead to false conclusions [24–27]. This is partially because “Failure to account for correlation is likely to underestimate the precision of a study, that is, to give it confidence intervals that are too wide and a weight that is too small” [27]. Meta-analyses based on aggregate (i.e., group mean) data are also susceptible to other sources of error, e.g., ecological bias [25].

Accordingly, we performed a systematic review and meta-analysis of the literature to determine the effects of ingesting dietary NO_3_^−^ on maximal muscle power in humans, as assessed using a randomized, double-blind, placebo-controlled, crossover experimental design. To account for the typically very high within-subject reproducibility of tests of human neuromuscular power (e.g., *r* = 0.99 [28];), we based our meta-analysis on the responses of individual subjects to NO_3_^−^ vs. placebo, as opposed to using aggregate data.

## Methods

The protocol for this study was registered at the International Prospective Register of Systematic Reviews (PROSPERO) and can be accessed at www.crd.york.ac.uk/PROSPERO/display_record.asp?ID=CRD42021238851. Conduct and reporting of the study was performed based on published guidelines [25, 27, 29, 30].

### Information sources

We conducted a systematic search of the following databases from their inception until May 4, 2021: Medline (Ovid), EMBASE (Embase.com), SportDiscus (EBSCO), Scopus (Scopus.com), Clinicaltrials.gov, and Google Scholar. For Google Scholar, all articles (excluding books) were retrieved from the first five pages. We did not restrict the search by language or publication date. Abstracts as well as full publications were included to minimize the potential for publication bias [31].

### Search strategy

The database search strategies were developed by a health sciences librarian (RJH) with expertise in literature searches. Known, relevant articles collected by the authors were analyzed to select keywords and subject headings. An initial search strategy in Medline Ovid was then iteratively developed by adding and removing additional keywords and subject headings until all known, relevant articles were retrieved by the search, and no new, relevant articles were found. The final search terms incorporated subject headings and keywords associated with dietary NO_3_^−^ and skeletal muscle power. The full search strategies for all information sources are provided in Supplemental Table 1.

The above automated searches were supplemented by a manual search of the literature, relying on the authors’ knowledge of existing publications, consultation of reference lists, etc. This searching turned up two additional publications.

### Inclusion and exclusion criteria

Studies were included if they 1) utilized a randomized, double-blind, placebo-controlled, crossover experimental design, 2) measured the effects of dietary NO_3_^−^ supplementation on peak or maximal neuromuscular power (in either watts or watts/kg body mass) performed with either a small or large muscle mass in the non-fatigued state (i.e., no immediately-prior exercise trials) in a normoxic, temperate environment, and 3) the within-subject correlation could be determined from data in the published manuscript or obtained from the authors. Studies not meeting these criteria (e.g., [32–40]) were excluded. For example, the studies of Corry and Gee [36] and Tatlichi and Çakmakçi [38] were excluded because they were not double-blind, whereas that of Williams et al. [39] was excluded because power was measured at 70% of one repetition-maximum (1 RM), which is far removed from the optimal resistance of < 30% of 1 RM for determination of peak or maximal muscle power [41]. The studies of Kokkinoplitis and Chester [35] and Conger, Zamzow, and Darnell [40] were excluded because we were unable to obtain the required data from the authors. The implications of excluding these two studies are considered in the Discussion.

**Fig. 1:**
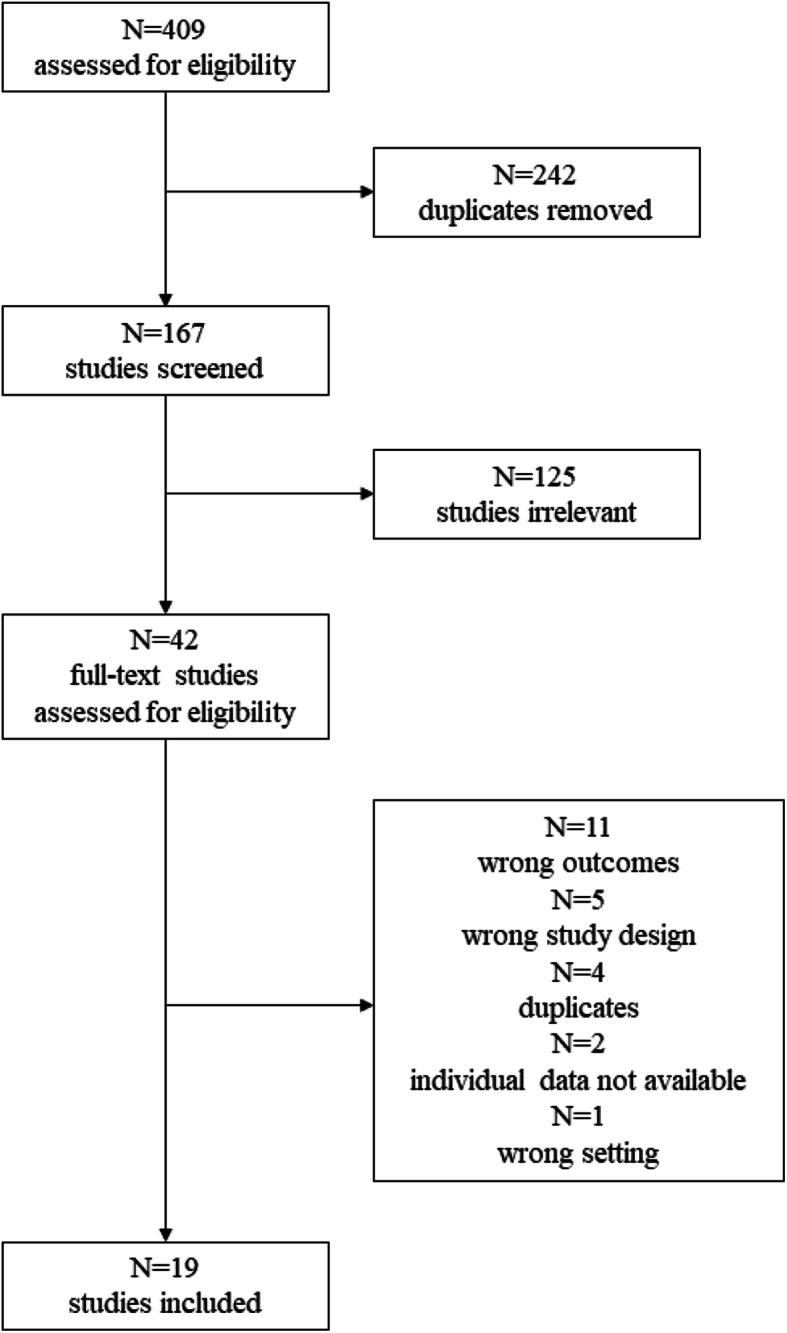
Preferred Reporting of Items for Systematic Reviews and Meta-Analyses (PRISMA) flow diagram

The Preferred Reporting of Items for Systematic Reviews and Meta-Analyses (PRISMA) flow diagram is shown in Fig. 1.

### Study selection and data extraction

All records were imported into Covidence software (Veritas Health Innovation) for de-duplication and screening. Screening was completed in two parts. First, titles and abstracts were independently screened by two authors (ARC and SJC) to determine their eligibility. Any disagreements were resolved in consultation with a third author (MNB). Second, full-text articles were screened using the same process. Data extraction was then independently performed by the same three authors. Specifically, study design, sample size, subject characteristics, form, dose, and duration of NO_3_^−^ supplementation, placebo characteristics, experimental procedures, and testing conditions were obtained from the text of eligible papers [42–60]. Effect sizes (ES), i.e., Hedge’s g, for NO_3_^−^-induced changes in peak or maximal power were calculated from either 1) the means and SDs for the NO_3_^−^ and placebo trials along with the exact *P* values for the within-subject comparison [49, 53, 55, 58, 60], or 2) anonymized individual subject data obtained from the authors [42–48, 50–52, 54, 56, 57, 59]. Since data from the 12 subjects in our original study [43] were also included in a subsequent report [50], only the results from the eight additional subjects in the latter study were used in the meta-analysis. A number of the studies employed a repeated sprint cycling protocol and/or only reported the mean power during a Wingate-style test [51, 52, 54, 55, 60]. In such instances, peak power from the first sprint was obtained from the authors and utilized in the meta-analysis. Since Jonvik et al. [52] found no difference between recreational, national caliber, and Olympic level speedskaters in the response to dietary NO_3_^−^, data from this study were treated as arising from a single pool of subjects. To avoid possible confounding effects from varying environmental conditions, only the data from the temperate trial in the study by Smith et al. [54] were used. For the study of Rodríguez-Fernández et al. [58], in which subjects performed concentric and eccentric squat exercises against varying inertial loads, the concentric peak power at the lowest load was used. Plotting of peak power against inertial load demonstrated that this resistance was close to optimal for determination of maximal muscle power. Finally, since we have recently shown that there is no beneficial effect of dietary NO_3_^−^ on muscle power at very high doses (i.e., 400 μmol/kg, or on average 27.4 mmol) [59], only the data from the low dose (i.e., 200 μmol/kg, or 13.7 mmol) trial of this study were used. (Why the beneficial effect of dietary NO_3_^−^ on muscle contractile function is diminished or even completely lost at very high doses is unclear. We have hypothesized, however, that it may be due to competition between NO_3_^−^ and NO_2_^−^ for reduction by xanthine oxidoreductase [61].)

### Quality assessment

Although numerous tools exist for judging the quality of randomized trials, most lack evidence of reliability and validity [30]. Based on the information extracted from each publication as described above, the quality of the studies was therefore judged against 13 elements of experimental design and execution held to be important in the context of the research question of interest [30]. Where uncertainties existed, authors were contacted for further clarification. Studies were classified as being of low (i.e., ≤8), moderate (i.e., 9–11), or high (i.e., ≥12) quality based on the presence or absence of these elements. The complete results of this quality assessment are included in Supplemental Table 2.

### Statistical analyses

For each individual study, Hedge’s g with small sample bias correction was calculated from the individual mean difference *d*_*i*_, the corresponding standard deviation *SD*_*i*_, and the sample size *N*_*i*_ as [62]:

$$ {g}_i=\left[1-\frac{3}{4\left({N}_i-1\right)-1}\right]\frac{d_i}{SD_i} $$

Its variance (var(*g*_*i*_)) was calculated as

$$ \operatorname{var}\left({g}_i\right)={\left[1-\frac{3}{4\left({N}_i-1\right)-1}\right]}^2\left(\frac{1}{N_i}\right)\left(\frac{N_i-1}{N_i-3}\right)\left(1+{N}_i{\delta}^2\right)-{\delta}^2, $$

where *δ* is the simple arithmetic mean of *g*_*i*_ [63]. The 95% confidence interval (CI) was obtained from the method of noncentral t distributions [64]. Both fixed-effects meta-analyses and random-effects meta-analyses were performed, with the primary ones used for interpretations decided based on the between-study heterogeneity evaluations. Heterogeneity between studies was evaluated by I^2^, H^2^, and the corresponding *p*-value. Funnel plot and Egger’s test were used to evaluate potential publication biases. Meta-regressions were used to assess the effects of a factor on the combined effect size. Given the limited study number, simple meta-regressions were used. Subgroup analyses were generated when needed. The 95% CIs of Hedge’s g and the forest and funnel plot were generated with Matlab R2020b (Mathworks, Natick, MA, USA). The meta-analyses were performed with R package metafor version 2.4–0 [62]. Two-sided *p*-values < 0.05 were considered statistically significant.

## Results

### Characteristics of included studies

We were able to identify and obtain data from 19 investigations that included a total of 268 subjects (218 men, 50 women). Selected characteristics of these studies are shown in Table 1. The dose of NO_3_^−^ used ranged from 6.4 (estimated) to 15.9 (measured) mmol, almost always (i.e., 17/19 studies) in the form of concentrated BRJ usually (i.e., 14/19 studies) given as an acute dose. The majority (i.e., 16/19) of the studies were judged to be of at least moderate quality, on average satisfying 11 ± 2 (SD) out of 13 of our pre-established criteria (including use of a randomized, double-blind, placebo-controlled, crossover design). The most common weaknesses identified were 1) failure to measure the actual dose of NO_3_^−^ administered (11/19) and 2) failure to measure any markers of resulting changes in NO_3_^−^ bioavailability (i.e., plasma NO_3_^−^ and NO_2_^−^, breath NO) (10/19). Several of the studies also used colored and flavored water instead of NO_3_^−^-depleted BRJ as a placebo, which was judged to inferior to the nitrate-depleted BRJ beverage used in the majority of studies (13/19).

**Table 1:** Characteristics of randomized, double-blind, placebo-controlled, crossover studies included in meta-analysis

Authors and year	Subjects		NO_3_^−^ dose					
	N	Age (y)	Source	Amount (mmol)				
				Measured	Assumed	Duration	Placebo	Test modality
Rothwell and Alkhatib 2014 [42]	12	21	BRJ		6.4	Acute dose	Other^a^	Sprint cycling
Coggan et al. 2015 [43]	12	36	BRJ	11.2		Acute dose	NO_3_^−^-free BRJ	Isokinetic knee extension
Coggan et al. 2015 [44]	9	57	BRJ	11.2		Acute dose	NO_3_^−^-free BRJ	Isokinetic knee extension
Rimer et al. 2016 [45]	13	26	BRJ	11.2		Acute dose	NO_3_^−^-free BRJ	Sprint cycling
Porcelli et al. 2016 [46]	7	25	High NO_3_^−^ diet		8.2	6 days	Normal NO_3_^−^ diet	Sprint cycling
Kramer et al. 2016 [47]	12	23	KNO_3_		8.0	6 days	KCl	Sprint cycling
Wylie et al. 2016 [48]	10	21	BRJ	8.2		5 days	NO_3_^−^-free BRJ	Sprint cycling
Domínguez et al. 2017 [49]	15	21	BRJ		6.4	Acute dose	Other^b^	Sprint cycling
Coggan et al. 2018 [50]	8	65	BRJ	11.2		Acute dose	NO_3_^−^-free BRJ	Isokinetic knee extension
Bender et al. 2018 [51]	12	17	BRJ		12.9	Acute dose	NO_3_^−^-free BRJ	Sprint cycling
Jonvik et al. 2018 [52]	52	24	BRJ		12.9	6 days	NO_3_^−^-free BRJ	Sprint cycling
Cuenca et al. 2018 [53]	15	22	BRJ		6.4	Acute dose	NO_3_^−^-free BRJ	Sprint cycling
Smith et al. 2019 [54]	12	22	BRJ		6.4	Acute dose	NO_3_^−^-free BRJ	Sprint cycling
Jodra et al. 2020 [55]	15	23	BRJ		6.4	Acute dose	NO_3_^−^-free BRJ	Sprint cycling
Coggan et al. 2020 [56]	12	71	BRJ	13.4		Acute dose	NO_3_^−^-free BRJ	Isokinetic knee extension
Jonvik et al. 2020 [57]	15	25	BRJ	15.9		6 days	NO_3_^−^-free BRJ	Isokinetic knee extension
Rodriguez-Fernandez et al. 2020 [58]	18	23	BRJ		12.9	Acute dose	Other^b^	Inertial load squats
Gallardo et al. 2021 [59]	9	70	BRJ	13.7		Acute dose	NO_3_^−^-free BRJ	Isokinetic knee extension
Dumar et al. 2021 [60]	10	20	BRJ		6.4	Acute dose	Other^c^	Sprint cycling

*BRJ* beetroot juice. ^a^Water colored and flavored with blackcurrant juice. ^b^Water colored and flavored with BRJ powder (1 g/L) and lemon juice. ^c^Undiluted blackcurrant juice

### Overall effect size

**Fig. 2:**
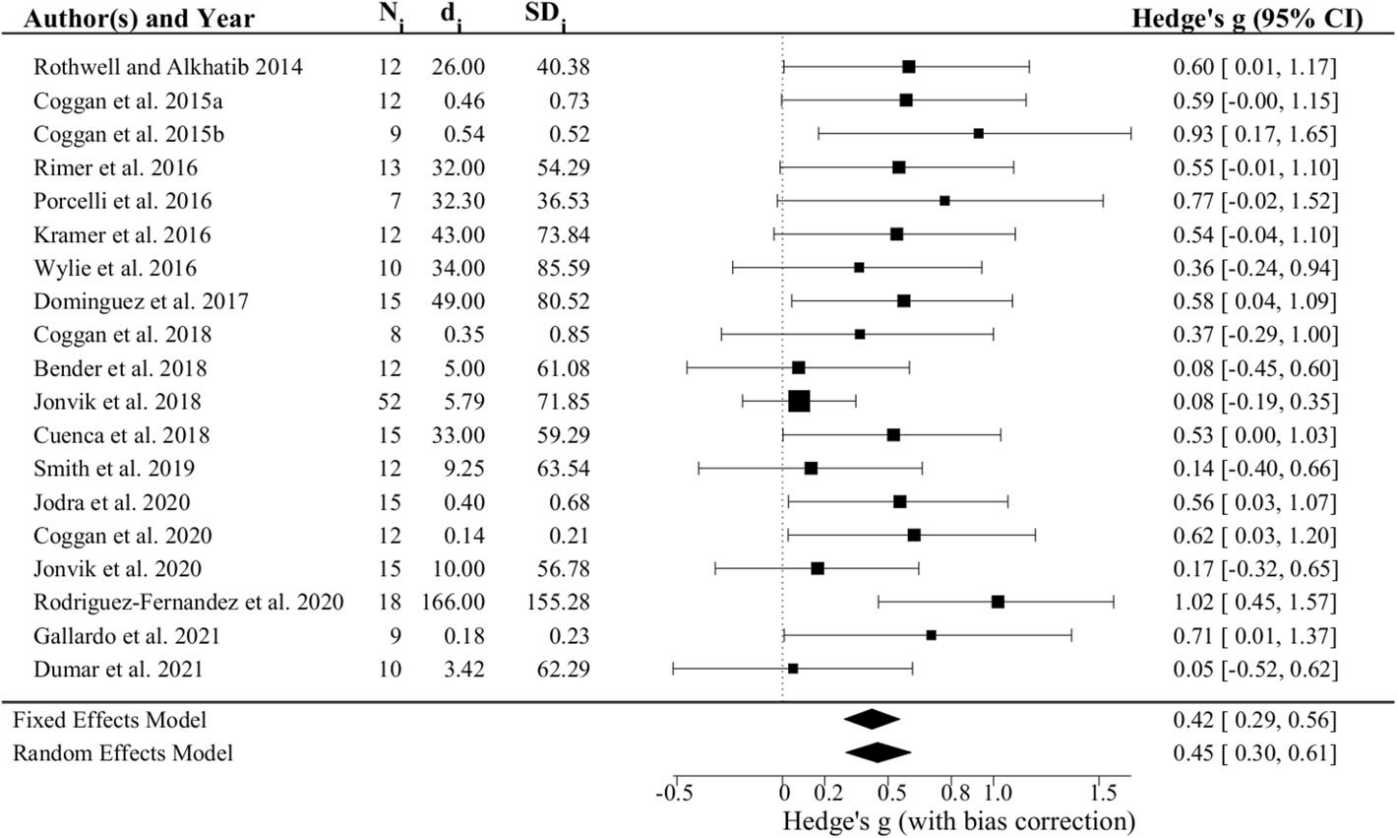
Forest plot of effect sizes (i.e., Hedge’s g) for all 19 studies of the effects of dietary NO_3_^−^ supplementation on maximal muscle power included in the meta-analysis. The size of each symbol is proportional to the weighting of each study, with the horizontal error bar representing the 95% confidence interval (CI). The horizontal diamonds at the bottom of the figure illustrate the overall effect size plus or minus the 95% CI as determined using fixed or random effects models

Although the magnitude varied somewhat and did not always achieve statistical significance, a positive effect of dietary NO_3_^−^ on muscle power was observed in 19/19 studies (Fig. 1). The overall ES as estimated using a fixed effects model was 0.42 (95% confidence interval (CI) 0.29, 0.56; *p* = 6.310 × 10^− 11^). There was limited heterogeneity between studies (i.e., I^2^ = 22.79%, H^2^ = 1.30, *p* = 0.3460). As a result, the ES estimated using a random effects model was similar (i.e., 0.45, 95% CI 0.30, 0.61; *p* = 1.064 × 10^− 9^; Fig. 2).

### Evaluation for publication bias/small study effects

**Fig. 3:**
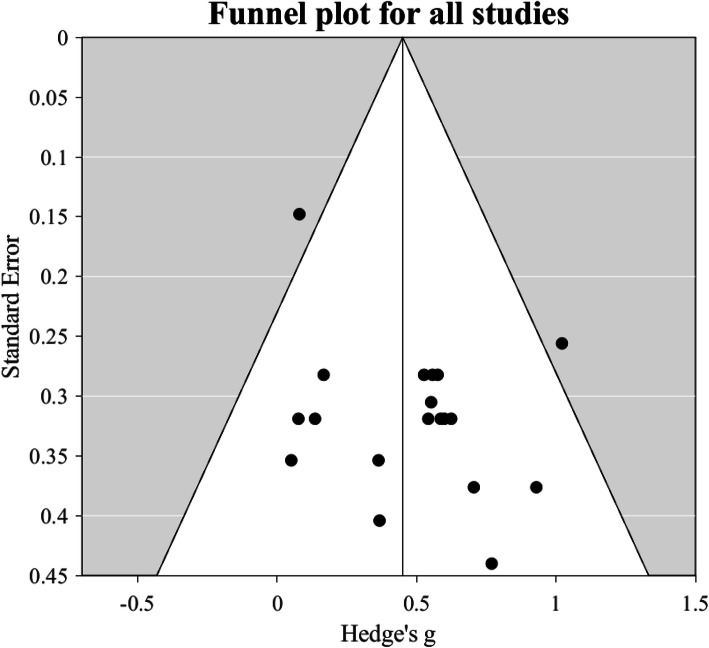
Funnel plot of standard error versus effect size (i.e., Hedge’s g) for all 19 studies of the effects of dietary NO_3_^−^ supplementation on maximal muscle power included in the meta-analysis

The funnel plot is shown in Fig. 3. Egger’s regression test for funnel plot asymmetry was not significant (*p* = 0.0780), arguing against any major publication bias/small study effects.

### Results of sub-group analyses

**Fig. 4:**
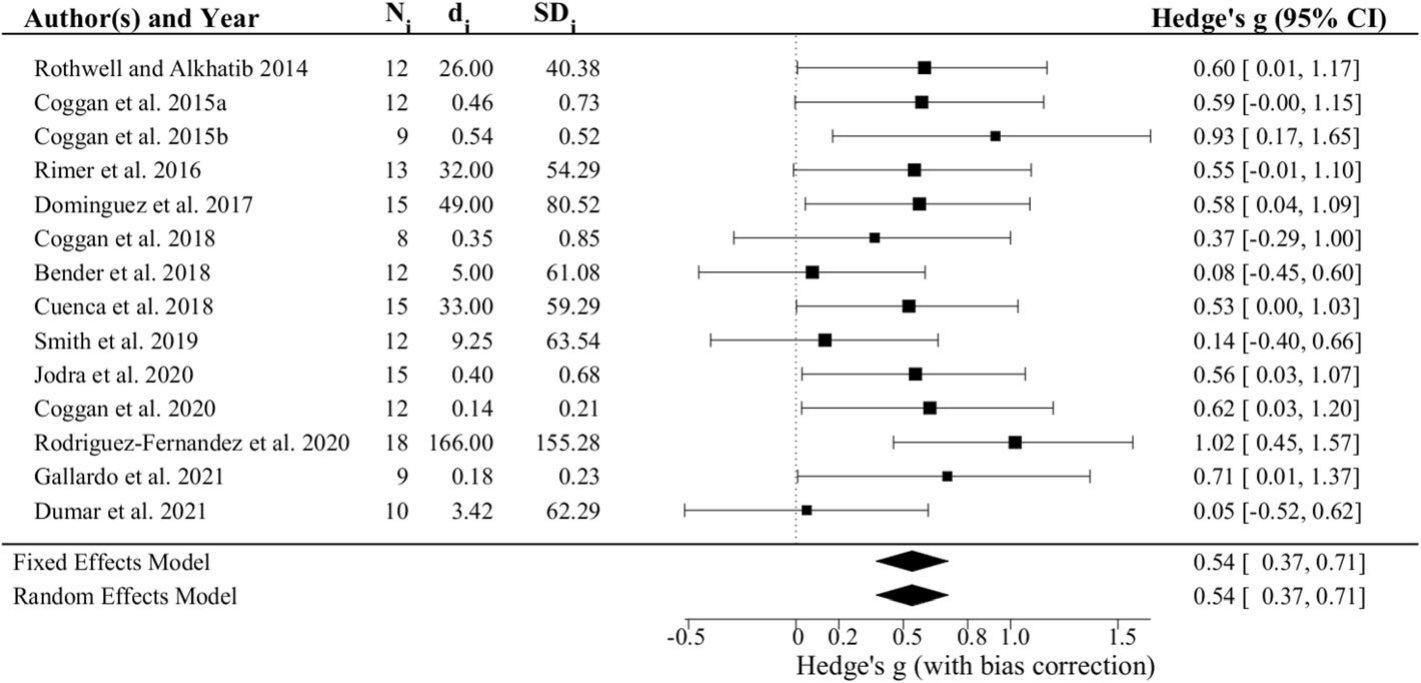
Forest plot of effect sizes (i.e., Hedge’s g) for the 14 studies of the effects of acute dietary NO_3_^−^ supplementation on maximal muscle power. See legend to Fig. 2 for additional details

**Fig. 5:**
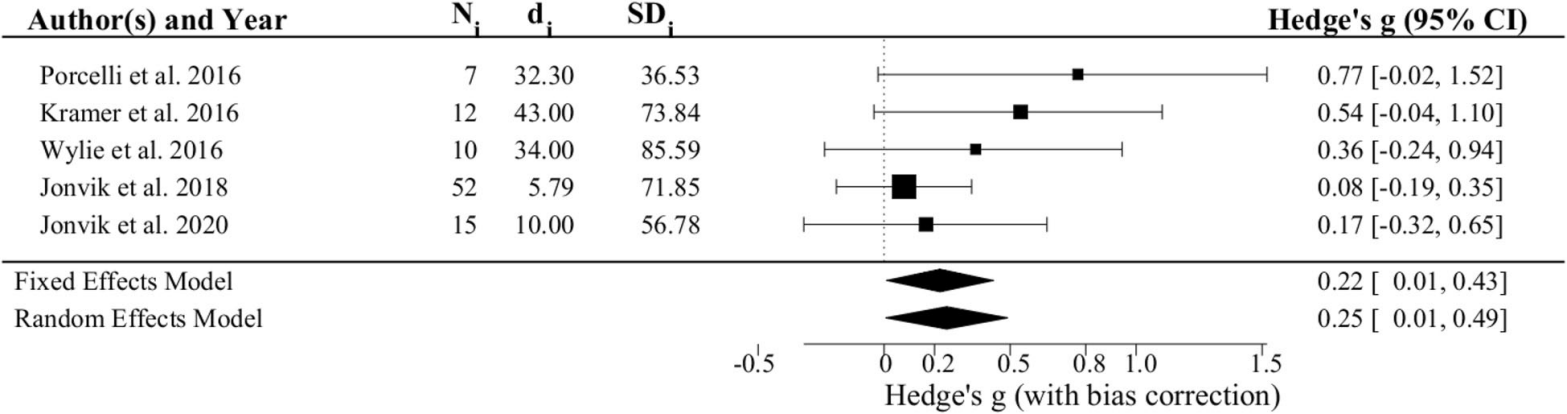
Forest plot of effect sizes (i.e., Hedge’s g) for the 5 studies of the effects of 5–6 d of dietary NO_3_^−^ supplementation on maximal muscle power. See legend to Fig. 2 for additional details

Exploratory sub-group analyses and meta-regressions were performed to evaluate the influence of subject age, sex, test modality (i.e., small versus large muscle mass exercise), and dietary NO_3_^−^ dosing regimen (i.e., acute dose vs. 5–6 d of treatment) on the ES. The effects of age, sex, and test modality were not significant (i.e., *p* = 0.1952, 0.9488, and 0.5328, respectively). In contrast, the ES from studies employing an acute NO_3_^−^ dose as estimated using a fixed effects model (i.e., 0.54, 95% CI 0.37, 0.71; *p* = 6.774 × 10^− 12^; Fig. 4) was 0.32 ± 0.14 greater (*p* = 0.0211) than that found in studies using a multi-day dosing regimen (i.e., 0.22, 95% CI 0.01, 0.43; *p* = 0.003630; Fig. 5). There was very little heterogeneity in either sub-grouping (i.e., I^2^ = 0.00%, H^2^ = 1.00, *p* = 0.6480 for acute dose studies and I^2^ = 12.26%, H^2^ = 1.14, *p* = 0.4211 for multiple dose studies). Nearly identical results were therefore obtained using a random effects model (Figs. 4 and 5).

## Discussion

This is the first systematic review and meta-analysis evaluating the effects of dietary NO_3_^−^ supplementation on maximal neuromuscular power in humans, and also the first individual participant data meta-analysis of the effects of dietary NO_3_^−^ on any aspect of human performance. Our primary finding is that NO_3_^−^ intake significantly enhances muscle power, thus supporting the conclusions of previous narrative reviews [14, 17]. This effect was statistically independent of subject age or sex and was evident during both small and large muscle mass exercise. Somewhat unexpectedly, however, the ES was greater in studies using an acute dose of NO_3_^−^ versus a multi-day supplementation protocol, although the latter was still significant.

As discussed by Riley et al. [25], performing meta-analysis using individual participant data provides numerous advantages, including the ability to account for within-subject correlation of results and the opportunity to perform subgroup analyses that may not be possible when using aggregate data. The former increases the power to detect treatment effects and is especially relevant in the present context, in which the average within-subject correlation of power during the placebo and NO_3_^−^ trials was 0.94 (range 0.73–0.99). Taking the latter into consideration, we found an overall ES of 0.43 (0.45 using a random effects model), which is substantially larger than those previously calculated from aggregate data for the effects of dietary NO_3_^−^ on either endurance time trial performance (i.e., ES = 0.05–0.12; 2–7) or on strength (i.e., ES = 0.08; 12). More importantly, however, an ES of this magnitude–which equates to a ~ 5% increase in maximal muscle power–is likely to be of considerable athletic and clinical significance, as previously discussed [43, 44, 56]. For example, it has been estimated that a mere 1% improvement in performance can double the odds of victory for an elite athlete [65]; in this context a 5% increase is enormous. Similarly, based on data from de Moura et al. [66] a 5% increase in maximal knee extensor power would be expected to reduce the odds of limitations in dynamic balance, habitual gait speed, or chair stand performance in inactive elderly subjects by roughly one-third. Thus, it is clear that dietary NO_3_^−^ holds considerable potential as a means of enhancing muscular power in both athletic and patient populations alike.

Some previous meta-analyses of the effects of dietary NO_3_^−^ on exercise performance have indiscriminately pooled data from studies utilizing disparate exercise tasks with markedly different physiological demands [4–7]. In contrast, we focused specifically on the novel outcome of maximal neuromuscular power. Perhaps as a result, we found only limited heterogeneity across studies. Nevertheless, we performed exploratory sub-group analyses to identify potential factors that might impact the ES. As stated above, subject age did not have a significant effect, which is in keeping with our prior small correlational study of 20 subjects [50]. Similarly, subject sex did not have a significant influence. This contrasts with the results of our previous investigation [50], in which the women (*n* = 7) appeared more likely to benefit from NO_3_^−^ supplementation. However, it also differs from the recent meta-analysis by Senefeld et al. [6], who concluded based on aggregate data (in which only 5% of studies were exclusively of women) that NO_3_^−^ supplementation does not enhance exercise performance in women. The reason for this apparent discrepancy is not known, but it may reflect the much larger number of women (and men) represented in the present meta-analysis. Finally, no difference was found between studies using small versus large muscle mass exercise (e.g., knee extension vs. cycling), a finding which parallels our previous work (e.g., 43 vs 45). Together, these findings support the robust nature of the effect of dietary NO_3_^−^ on human muscle power.

In contrast to the lack of effect of age, sex, and muscle mass, the dietary NO_3_^−^ dosing regimen used may have an impact. Specifically, studies using a multi-day supplementation protocol exhibited a lower (but still significant) ES compared to studies employing an acute dose of NO_3_^−^. One possible explanation for this finding is that repeated dosing exceeds the optimal NO_3_^−^ concentration for enhancing contractile function in skeletal muscle [59]. However, pharmacokinetic modeling of the combined NO_3_^−^/NO_2_^−^ system indicates that there should be little further accumulation in tissue after the first couple of doses [67]. Furthermore, prior meta-analyses of the effects of dietary NO_3_^−^ on endurance exercise performance have revealed no differences between acute and chronic dosing regimens [3–6]. Thus, additional research is required to determine whether the acute NO_3_^−^-induced improvement in muscle power is in fact diminished with repeated dosing, especially in patient populations who might benefit from NO_3_^−^ supplementation during activities of daily living (vs. athletes who might ingest NO_3_^−^ only prior to competitions).

As is true with endurance exercise [3, 5, 6], other potential modulators of the effects of dietary NO_3_^−^ on muscle power include the actual dose of NO_3_^−^ ingested [59] and/or the plasma levels of NO_3_^−^ and/or NO_2_^−^ attained following NO_3_^−^ ingestion [50]. We did not attempt to assess these factors in the present meta-analysis, however, because less than half of the included studies directly measured either of these parameters, and aside from our studies [43, 44, 50, 56, 59] only Wylie et al. [48] and Jonvik et al. [57] measured both. This is a significant weakness of the current literature in this area, as we have found the NO_3_^−^ content of various BRJ supplements commonly used in such studies can vary considerably [68], even when obtained from the same manufacturer. Furthermore, we have found that the NO_3_^−^ content of various BRJ products decreases gradually over time (unpublished observations). Future studies should therefore explicitly document the precise dose of NO_3_^−^ provided as well as quantify the impact this has on biomarkers of NO bioavailability.

Based on previous research of the effects of NO_3_^−^ supplementation on endurance exercise performance [2, 4–6], another potential modulator of the ES among subjects is their training status or level of physical activity. We did not formally attempt to assess this possible influence, due in part to the difficulty of objectively classifying subjects in the various studies, many of whom were simply described as being “recreationally active”. As previously noted, however, we found only limited statistical heterogeneity across investigations, even though the subjects ranged from patients with heart failure [44] to healthy older individuals [56, 59] to Olympic-level athletes [52]. This argues against subject population having a major impact on the magnitude of the dietary NO_3_^−^-induced increase in muscle power. Nonetheless, it is interesting to note that two of the three studies exhibiting the smallest ES (i.e., [52, 60]) tested highly trained sprinters. Additional research is therefore warranted to determine whether regular sprint training attenuates the positive effects of NO_3_^−^ supplementation on maximal power.

Regrettably, we were unable to obtain the data required to include additional studies by Kokkinoplitis and Chester [35] and Conger, Zamzow, and Darnell [40] in our meta-analysis. However, conservatively assuming the same within-subject correlation as the lowest found in the included studies (i.e., 0.73), the ES in the *n* = 7 subjects studied by Kokkinoplitis and Chester [35] would have been 0.36 (95% CI -0.33, 1.02), which is similar to that calculated from the data in the present meta-analysis. Applying the same logic to the results from the *n* = 14 subjects studied by Conger, Zamzow, and Darnell [40] extracted from their Fig. 1a yields a lower ES, i.e., 0.13 (95% CI -0.37, 0.62). This may be because these authors used a BRJ powder that we have found to contain very little NO_3_^−^ [68]. Regardless, even if the individual subject data had been available the inclusion of either or both of these two additional studies would have been unlikely to have appreciably changed our results, which are based on 19 other studies of a total of 268 subjects.

Although the results of the present meta-analysis clearly demonstrate that dietary NO_3_^−^ increases muscle power in humans, the mechanism responsible for this effect still remains to be established. We have previously hypothesized that it may be due to greater NO-stimulated production of cyclic guanosine monophosphate (cGMP), leading to increased myofibrillar Ca^2+^ sensitivity as a result of enhanced phosphorylation of the myosin regulatory light chain (RLC) [17]. Recently, though, Kumar et al. [69] reported that NO_3_^−^ supplementation did not increase RLC phosphorylation in the diaphragm of aged mice, even though it did increase peak power. As discussed previously [17], there are notable differences between rodents and humans in dietary NO_3_^−^/NO_2_^−^/NO metabolism, making the relevance of these findings unclear. It is also possible that dietary NO_3_^−^ increases Ca^2+^ sensitivity and hence muscle power via a non-cGMP-dependent mechanism, e.g., increased nitros(yl)ation of the ryanodine receptor [17]. As such, the biochemical mechanism by which NO_3_^−^ intake improves human muscle power therefore requires additional study.

## Conclusions

The present meta-analysis lends quantitative support to previous narrative reviews [14, 17] that have concluded that NO_3_^−^ supplementation enhances maximal neuromuscular power in humans. Based on the currently available literature, this ergogenic effect is seemingly independent of subject age, sex, or the amount of muscle mass engaged in the activity but may be greater with acute vs. repeated dosing. Importantly, this dietary NO_3_^−^-induced increase in power is sufficient to have important practical and clinical implications. Further research to determine the optimal supplementation regimen, target population, etc., is therefore imperative.

## Supplementary Information

**Additional file 1: Supplemental Table 1.** Database search strategies.

**Additional file 2: Supplemental Table 2.** Quality assessment of studies included in meta-analysis.

## Data Availability

The datasets used and/or analyzed during the current study are available from the corresponding author on reasonable request.

## Abbreviations

1 RM: One repetition maximum
BRJ: Beetroot juice
KNO_3_^-^: Potassium nitrate
NO: Nitric oxide
NO_2_^-^: Nitrite
NO_3_^-^: Nitrate
O_2_: Oxygen
PRISMA: Preferred Reporting of Items for Systematic Reviews and Meta-Analyses
