# Supplementary material for: Effect of dietary nitrate on human muscle power: a systematic review and individual participant data meta-analysis
Source: J Int Soc Sports Nutr. 2021 Oct 9;18:66. doi: 10.1186/s12970-021-00463-z (PMC8501726; doi:10.1186/s12970-021-00463-z)
Supplement: Supplementary file 1 — Additional file 1: Supplemental Table 1. Database search strategies. [file 12970_2021_463_MOESM1_ESM.docx]

Supplemental Table 1. Database search strategies.

| Database | Search parameters |
| --- | --- |
| Medline Ovid | (Beta vulgaris/ OR ((Nitrates/ OR Nitric Oxide/) AND (Muscle, Skeletal/ OR Muscle Fibers, Skeletal/)) OR (Beet OR beets OR beetroot* OR (nitrate* adj5 (diet* OR consum*))).tw.) AND ((Power adj10 (muscul* OR Muscle* OR neuromuscular OR bench* OR barbell* OR press OR extensor OR output)).tw OR ("isokinetic dynamometry" OR Wingate OR "inertial load" OR "inertial cycling" OR "vertical jump" or "countermovement jump" or "squat jump" or plyometrics or "drop jump" or "depth jump" or "jump training" OR "leg press" OR "leg rig" OR "Nottingham Power Rig").tw.) |
| Embase | ('beetroot juice'/de OR 'beet'/exp OR (('nitrate'/de OR 'nitric oxide'/de) AND ('skeletal muscle'/exp OR 'skeletal muscle cell'/de)) OR beet:ab,ti,kw OR beets:ab,ti,kw OR beetroot*:ab,ti,kw OR (nitrate* NEAR/5 (dietary OR consum*)):ab,ti,kw) AND (‘isokinetic dynamometry'/de OR 'wingate test'/de OR (Power NEAR/10 (muscul* OR Muscle* OR neuromuscular OR bench* OR barbell* OR press OR extensor OR output)):ab,ti,kw OR ("isokinetic dynamometry" OR Wingate OR "inertial load" OR "inertial cycling" OR "vertical jump" or "countermovement jump" or "squat jump" or plyometrics or "drop jump" or "depth jump" or "jump training" OR "leg press" OR "leg rig" OR "Nottingham Power Rig"):ab,ti,kw) |
| SPORTDiscus | (DE "BEETS" OR DE "BEET juice” OR ((DE "NITRATES" OR DE "NITRIC oxide") AND (DE "SKELETAL muscle")) OR beet OR beets OR beetroot* OR (nitrate* N5 (dietary OR consum*))) AND (DE "MUSCLE strength" OR DE "WINGATE Anaerobic Test" OR (Power N10 (muscul* OR Muscle* OR neuromuscular OR bench* OR barbell* OR press OR extensor OR output)) OR "isokinetic dynamometry" OR Wingate OR "inertial load" OR "inertial cycling" OR "vertical jump" or "countermovement jump" or "squat jump" or plyometrics or "drop jump" or "depth jump" or "jump training" OR "leg press" OR "leg rig" OR "Nottingham Power Rig")  Limits: Academic Journals |
| Scopus | (TITLE-ABS (beet OR beets OR beetroot* OR (nitrate* W/5 (dietary OR consum*))) AND  TITLE-ABS ((Power W/10 (muscul* OR Muscle* OR neuromuscular OR bench* OR barbell* OR press OR extensor OR output)) OR "isokinetic dynamometry" OR Wingate OR "inertial load" OR "inertial cycling" OR "vertical jump" or "countermovement jump" or "squat jump" or plyometrics or "drop jump" or "depth jump" or "jump training" OR "leg press" OR "leg rig" OR "Nottingham Power Rig") |
| Clinicaltrials.gov | Intervention/treatment: beet OR beets OR beetroot* OR “dietary nitrate” OR “nitrate supplement” OR “nitrate supplementation Outcome: “muscle power” OR “muscular power” OR “neuromuscular power” OR “bench press” OR “power output” OR "isokinetic dynamometry" OR Wingate OR "inertial load" OR "inertial cycling" OR "vertical jump" or "countermovement jump" or "squat jump" or plyometrics or "drop jump" or "depth jump" or "jump training" OR "leg press" OR "leg rig" OR "Nottingham Power Rig" |
| Google Scholar | Beet*\|beetroot*\|nitrate muscle*\|muscular power\|wingate\|"isokinetic dynamometry" Download first 5 pages, except for books and citation-only items |
