# Supplementary material for: Effect of dietary nitrate on human muscle power: a systematic review and individual participant data meta-analysis
Source: J Int Soc Sports Nutr. 2021 Oct 9;18:66. doi: 10.1186/s12970-021-00463-z (PMC8501726; doi:10.1186/s12970-021-00463-z)
Supplement: Supplementary file 2 — Additional file 2: Supplemental Table 2. Quality assessment of studies included in meta-analysis. [file 12970_2021_463_MOESM2_ESM.docx]

Supplemental Table 2. Quality assessment of studies included in meta-analysis.

| Authors | Randomized? | Double-  blind? | Crossover? | Placebo-controlled? | True  placebo? | NO_3_^-^ dose measured? | NO_3_^-^/NO_2_^-^ measured? | NO  measured? | Diet  controlled? | Physical activity controlled? | Mouthwash use controlled? | Familiarzation  trial? | Time of day  controlled? | Overall  quality |
| --- | --- | --- | --- | --- | --- | --- | --- | --- | --- | --- | --- | --- | --- | --- |
| Rothwell and Alkhatib 2014 (42) | Y | Y | Y | Y | N | N | N | N | Y | Y | N | Y | Y | L |
| Coggan et al. 2015 (43) | Y | Y | Y | Y | Y | Y | Y | Y | Y | Y | Y | N | Y | H |
| Coggan et al. 2015 (44) | Y | Y | Y | Y | Y | Y | Y | Y | Y | Y | Y | N | Y | H |
| Rimer et al. 2016 (45) | Y | Y | Y | Y | Y | Y | N | N | Y | Y | Y | Y | Y | M |
| Porcelli et al. 2016 (46) | Y | Y | Y | Y | Y | N | Y | N | Y | Y | Y | Y | Y | M |
| Kramer et al. 2016 (47) | Y | Y | Y | Y | Y | N | N | N | Y | Y | Y | Y | Y | M |
| Wylie et al. 2016 (48) | Y | Y | Y | Y | Y | Y | Y | N | Y | Y | Y | Y | Y | H |
| Domínguez et al. 2017 (49) | Y | Y | Y | Y | N | N | N | N | Y | Y | Y | N | Y | L |
| Coggan et al. 2018 (50) | Y | Y | Y | Y | Y | Y | Y | Y | Y | Y | Y | N | Y | H |
| Bender et al. 2018 (51) | Y | Y | Y | Y | Y | N | N | N | Y | Y | Y | N | Y | M |
| Jonvik et al. 2018 (52) | Y | Y | Y | Y | Y | N | Y | N | Y | Y | Y | Y | Y | M |
| Cuenca et al. 2018 (53) | Y | Y | Y | Y | Y | N | N | N | Y | Y | Y | Y | Y | M |
| Smith et al. 2019 (54) | Y | Y | Y | Y | Y | N | N | N | Y | Y | Y | Y | Y | M |
| Jodra et al. 2020 (55) | Y | Y | Y | Y | Y | N | N | N | Y | Y | Y | N | Y | M |
| Coggan et al. 2020 (56) | Y | Y | Y | Y | Y | Y | Y | Y | Y | Y | Y | Y | Y | H |
| Jonvik et al. 2020 (57) | Y | Y | Y | Y | Y | Y | Y | N | Y | Y | Y | Y | Y | H |
| Rodriguez-Fernandez et al. 2020 (58) | Y | Y | Y | Y | N | N | N | Y | Y | N | Y | Y | Y | M |
| Gallardo et al. 2021 (59) | Y | Y | Y | Y | Y | Y | Y | Y | Y | Y | Y | Y | Y | H |
| Dumar et al. 2021 (60) | Y | Y | Y | Y | N | N | N | N | Y | Y | Y | N | Y | L |

L = low (≤8 elements met), M = moderate (9-11 elements met), H = high (≥12 elements met).
